# Direct hospital costs and cost drivers in Greek intensive care units: a micro-costing analysis

**DOI:** 10.62838/jccm-2026-0060

**Published:** 2026-10-30

**Authors:** Alkmena Kafazi, Maria Katharaki, Christos Stylianou, Pavlos Myrianthefs

**Affiliations:** Department of Nursing, National and Kapodistrian University of Athens, Athens, Greece; Cardiac Intensive Care Unit, The 417 Army Equity Fund Hospital, Athens, Greece

**Keywords:** micro-costing analysis, intensive care units, critical care, cost drivers, fixed cost, variable cost, nurse staffing, Greece

## Abstract

**Introduction:**

Intensive Care Units (ICUs), though comprising a small proportion of total hospital beds, represent a disproportionately high share of healthcare expenditure. Accurate cost assessment is vital for optimizing resource allocation, particularly in resource-limited settings like Greece.

**Aim of the study:**

To estimate the direct hospital cost of ICU hospitalization in adult patients using a micro-costing approach and to identify clinical and organizational factors driving these costs.

**Material and Methods:**

A prospective surveillance study was conducted over two years in four multidisciplinary adult ICUs in the Attica region. Detailed patient-level data on demographics, clinical status, interventions, and resource use were collected daily. Direct ICU costs, both fixed and variable, were calculated from the hospital perspective using a bottom-up micro-costing approach. Multivariable linear regression was used to identify independent predictors of ICU costs.

**Results:**

The analysis included 500 patients, representing 12,689 patient-days. The total hospitalization cost was €6.33 million. The median cost per ICU admission was €7,756.8, with variable costs comprising 72.5% of total expenditures. Antibiotics accounted for nearly 50% of variable costs. Significant independent cost drivers included blood transfusion (additional €2,063), prolonged ICU stay (each day added ~€264), ventilator-associated events (+€2,374), central line-associated bloodstream infections (+€1,877), surgical site infections (+€6,609), and patient-to-nurse ratios >2 (+€5,000).

**Conclusions:**

ICU costs are shaped not only by patient severity and interventions but also by modifiable structural factors such as infection rates and staffing ratios. Investments in infection prevention, evidence-based transfusion practices, and adequate nurse staffing could yield substantial cost savings while improving patient outcomes in Greek ICUs. These insights provide critical, evidence-informed guidance for strengthening critical care in resource-limited settings.

## Introduction

Although Intensive Care Units (ICUs) account for only approximately 10% of total hospital bed capacity, they are responsible for nearly one-third of total hospital expenditure [1,2]. Currently, ICU admissions represent approximately 11% of all hospitalizations, a proportion expected to rise further due to population aging and the increasing severity of illness among hospitalized patients [3]. The average daily cost of an ICU bed is nearly three times higher than that of a general ward bed, underscoring the resource-intensive nature of critical care services [4].

Health economists have long focused on identifying the key factors driving hospital expenditure growth. International organizations such as the World Health Organization (WHO) and the Centers for Disease Control and Prevention (CDC) have proposed various theoretical and econometric frameworks to analyze hospital care costs and their evolution over time [5,6].

The most commonly used approach to cost assessment in healthcare includes the categorization of costs into three major types: direct, indirect, and intangible [6]. Direct costs refer to expenditures directly related to patient care, such as salaries of medical and nursing staff, pharmaceuticals, and medical supplies. These are further subdivided into fixed and variable costs [6]. Fixed costs remain constant regardless of patient volume or occupancy rates and are typically associated with staff salaries and equipment maintenance; they can represent up to 80% of total ICU expenditures. Variable costs fluctuate based on hospital activity levels, including costs for drugs, consumables, and diagnostic procedures. Indirect costs capture the broader economic impact on society due to illness or premature death, such as lost productivity, reduced work capacity, and long-term disability. Intangible costs refer to qualitative and non-monetary aspects of disease burden, such as pain, psychological distress, social isolation, or stigma. These are particularly difficult to quantify but are essential to a comprehensive socioeconomic evaluation [6,7,8].

Costing methodologies in hospital settings, and particularly in ICUs, can be broadly classified into two main approaches: micro-costing and standard or average costing. Micro-costing (bottom-up) involves the detailed identification and measurement of all resources consumed by each individual patient. It is a highly accurate method, rooted in microeconomic analysis, but requires substantial time and expertise [9]. For ICU admissions, this includes itemized tracking of drug usage, personnel costs, diagnostic tests, and equipment utilization on a per-patient basis [6,10,11]. Standard or average costing (top-down), in contrast, involves dividing total hospital expenditures by the total number of patient admissions or bed-days. While simpler and more retrospective in nature, this approach assumes equal resource distribution across patients and is limited in comparing costs across different diseases and treatment protocols [6,10]. ICU patients exhibit extreme heterogeneity in resource consumption, with individual costs varying significantly depending on severity of illness and length of stay [11]. Accurately capturing this variance requires detailed micro-costing methodologies to account for patient-level resource intensity [9]. This variability renders top-down averages particularly unreliable in the critical care context, as they may mask clinically and economically meaningful differences between patient subgroups and obscure the specific drivers of expenditure. Micro-costing is therefore especially well-suited to ICU research, where substantial variability in patient severity, complications, and treatment intensity necessitates precise, patient-level measurement of resource utilization to accurately identify modifiable cost drivers and inform resource allocation [9]. Given the disproportionately high economic footprint of ICU care and the increasing demand for critical care services, accurate and detailed cost estimation is imperative. This allows healthcare systems to optimize resource allocation, assess cost-effectiveness of interventions, and inform policy decisions that balance clinical benefit with financial sustainability.

Despite the increasing economic burden of intensive care, detailed cost analyses based on micro-costing methodologies remain limited, particularly in middle-resource healthcare systems such as Greece [12]. Most existing studies rely on top-down approaches [12,13,14], which may obscure patient-level variability and limit the identification of key cost drivers. By enabling a more precise assessment of resource utilization, micro-costing provides a valuable framework for understanding ICU costs; however, its application in the Greek healthcare system remains scarce. The Greek National Health System operates under particular resource constraints, including staffing pressures and among the highest rates of antimicrobial resistance in European acute care settings (15), both of which are likely to shape the cost structure of critical care in ways that differ meaningfully from high-resource systems. Yet patient-level cost data specific to Greek ICUs and to the clinical and organizational factors driving those costs, remain largely unavailable, particularly from prospective, patient-level analyses that integrate both clinical and organizational cost drivers. To date, prospective multi-center micro-costing studies in this setting are limited, representing an important gap in the evidence base for critical care resource planning in Greece.

To address this gap, the aim of this study was to estimate the direct costs of ICU hospitalization in adult patients using a micro-costing approach and to identify the clinical and organizational factors associated with these costs.

## Methods

### Study design

A prospective surveillance study was conducted in four multidisciplinary adult Intensive Care Units (ICUs) in the Attica region of Greece over a two-year period. The ICUs were selected as a convenience sample based on feasibility of prospective data collection and institutional collaboration. All were large, tertiary-care public hospitals and broadly reflect the structural and operational characteristics of public-sector critical care in Greece, including staffing constraints and high patient acuity. The units were general medical–surgical ICUs, two of which were affiliated with public universities, with 9–15 beds and patient-to-nurse ratios ranging from 1:1 to 4:1. All operated under similar organizational structures within the Greek National Health System and followed standardized clinical protocols. Infection prevention and control programs, including routine surveillance of healthcare-associated infections, were in place in all units, although their intensity and resource allocation varied.

The study population included all adult patients admitted to the ICUs during the study timeframe. Patients were followed from the time of ICU admission until discharge or death. Individuals under the age of 18 and patients with an ICU length of stay <24h were excluded from the study, as such short admissions often represent transient monitoring or early transfers and do not reflect the full spectrum of ICU resource utilization, potentially leading to underestimation of true costs.

Patient data were systematically gathered on a daily basis from medical charts, nursing documentation, and microbiology laboratory records. For each patient, demographic and clinical information including age, sex, and reason for ICU admission was recorded. Severity of illness was assessed on the day of ICU admission using the APACHE II score and the McCabe classification for underlying disease prognosis. Complications such as sepsis, pressure ulcers, renal failure, and health-care-associated infections (HCAIs)—including ventilator-associated events (VAE), central line-associated bloodstream infections (CLABSI), catheter-associated urinary tract infections (CAUTI), and surgical site infections (SSI)—were also documented. The diagnosis of HCAIs was based on standard CDC definitions [16]. Additionally, the duration of mechanical ventilation, length of ICU stay, and patient outcome (ICU discharge or death) were recorded. Furthermore, daily documentation of the patient-to-nurse ratio was performed in each ICU. In cases where staffing levels varied throughout the day, the maximum ratio observed during any 24-hour period was used in the analysis.

The primary outcome of the study was the total direct ICU hospitalization cost per patient. Secondary outcomes included the identification of clinical and organizational factors associated with increased ICU costs, as well as the relative contribution of fixed and variable cost components.

To ensure data accuracy, all collected information was independently cross-checked by members of the research team blinded to clinical decision-making, using multiple data sources including medical charts, nursing records, and laboratory systems. In cases of missing or incomplete data, efforts were made to retrieve the information from primary records, and variables with irretrievable missing values were excluded from the respective analyses.

### Cost analysis

This analysis was conducted from the hospital perspective and included only direct ICU costs. All costs were calculated in Euros (€) based on official price lists corresponding to the year 2018. No inflation adjustment was applied, as unit costs were derived from standardized national sources.

The direct costs associated with ICU hospitalization were meticulously estimated employing a bottom-up costing approach, a microeconomic methodology that facilitates a comprehensive allocation of expenses at the individual patient level based on precise resource utilization [6,10]. For each patient, total direct hospital costs, encompassing both fixed and variable components, were rigorously calculated.

Fixed daily costs were derived from personnel remuneration and ICU operational and maintenance expenditures. These cost parameters were obtained from official datasets provided by the Greek Ministry of Health, based on the annual financial reports submitted by the participating institutions [17]. The length of stay within the ICU was prospectively documented for each patient, enabling the calculation of total fixed costs by multiplying the length of stay by the corresponding daily fixed ICU cost.

Variable costs comprised daily expenditures for antibiotics and other pharmacological agents, enteral and parenteral nutritional support, blood products, medical consumables, and diagnostic laboratory and imaging procedures. Medication costs were precisely calculated by multiplying the administered daily dose of each pharmaceutical agent by the duration of treatment per patient, followed by multiplication with the unit cost per dosage form (e.g., gram, milliliter). These cost data for pharmaceuticals, nutritional products, and blood transfusions were sourced from authoritative information published by the Ministry of Health [18].

Medical supply costs were obtained via the “Observe Net” platform, managed by the Health Supplies Committee, which functions as the official price observatory for medical consumables utilized in Greek public hospitals [19]. Supplies incorporated in the study included endotracheal and tracheostomy tubes, central venous and urinary catheters, pressure ulcer prevention pads, and devices used for enteral and parenteral feeding.

Costs for diagnostic procedures were acquired from the National Organization for Healthcare Services Provision’s official repository [20]. These expenses were multiplied by the number of diagnostic interventions performed per patient. The diagnostic modalities accounted for included electrocardiography, electroencephalography, conventional radiography, ultrasonography, computed tomography, magnetic resonance imaging, as well as microbiological cultures.

### Statistical analysis

Categorical variables were presented as absolute numbers (N) and relative frequencies (%), while continuous variables were expressed as medians and interquartile ranges (IQR). Normality was evaluated with the Kolmogorov–Smirnov test. Differences between groups were assessed using the non-parametric Mann–Whitney U test.

Factors influencing ICU hospitalization costs were investigated using a multivariate linear regression model. Following univariate linear regression analysis, variables with p-values < 0.05 were included in the multivariate model. Associations are reported as regression coefficients (B) with 95% confidence intervals (CI) and corresponding p-values. Additionally, the adjusted coefficient of determination (adjusted R^2^) is presented, indicating the proportion of variance in the dependent variable explained by the independent variables and serving as a measure of the model’s goodness of fit.

Prior to conducting multiple linear regression, multicollinearity among independent variables was examined. After building the multivariate linear regression model, diagnostic checks were performed for: (a) independence of observations, (b) presence of outliers, (c) linearity between the dependent and independent variables, (d) normality of residuals, and (e) homoscedasticity of residuals.

Since the distribution of the ICU cost variable was skewed, its natural logarithm was used as the dependent variable in the multivariable linear regression model. To facilitate interpretation, the regression coefficients were exponentiated (Exp(B) = eB), reflecting the multiplicative effect on the geometric mean of the original (non-logged) cost [21]. The additional cost associated with each predictor was estimated using the formula: additional cost = median cost × (Exp(B) – 1).

All analyses were performed using IBM SPSS Statistics package, version 22.0. All tests were two-sided, and statistical significance was set at p < 0.05 with a 95% confidence level.

### Research ethics

The present prospective research study adhered to the fundamental principles of ethics and research conduct. The data did not include sensitive information nor any information that could directly affect patient care. Full confidentiality was maintained for all collected data, anonymity was ensured, and the research protocol received approval from the Institutional Review Boards of the participating hospitals. Written informed consent was obtained from all the patients before their participation in this study.

## Results

During the two-year study period, 500 patients were admitted to four multidisciplinary ICUs in the Attica region, accumulating a total of 12,689 patient-days and 10,099 days of mechanical ventilation. The majority of patients were male (59.4%), with a median age of 61.5 years (Table 1).

The total cost of ICU care during the data collection period amounted to €6,329,228.3, with a median cost of €7,756.8 (IQR €3,272.6 – €16,095.0) per patient. Variable costs represented the highest component of the total expenditure, with antibiotics comprising nearly 50% of these costs (Table 2).

The total cost per ICU Day was €498.8 (calculated as total cost divided by total patient-days), with fixed costs amounting to €137.2 and variable costs totaling €361.6.

As shown in Table 3, the median ICU hospitalization cost was higher among male patients, those with APACHE II score ≥18, those with ultimately or rapidly fatal diseases, and patients admitted due to trauma or infection. Increased costs were also observed in patients with renal failure, those who received blood transfusions, and those diagnosed with sepsis, pressure ulcers, or HCAIs, including VAE, CLABSI, CAUTI, and SSI. Patients treated in ICU for ≥19 days, those who received mechanical ventilation for ≥15 days, those admitted to units with a patient-to-nurse ratio >2, and non-survivors also incurred significantly higher hospitalization costs.

Univariable analysis identified several factors significantly associated with increased ICU hospitalization cost, including male sex, higher APACHE II score, presence of ultimately or rapidly fatal disease, infection or trauma as reason for ICU admission, renal failure, sepsis, blood transfusion, pressure ulcers, HCAIs, prolonged ICU stay and mechanical ventilation, patient-to-nurse ratio >2, and death. In the multivariable analysis, independent predictors of increased ICU cost included blood transfusion (Exp(B) = 1.266, p<0.001), ICU length of stay (Exp(B) = 1.034, p<0.001), VAE (Exp(B) = 1.306, p<0.001), CLABSI (Exp(B) = 1.242, p=0.006), SSI (Exp(B) = 1.852, p=0.003), and patient-to-nurse ratio >2 (Exp(B) = 1.644, p<0.001) (Table 4).

**Table 1.** Patient demographic and clinical characteristics

**Variable**	**N (%) or Median (IQR)**
Age, years	61.5 (47–74)
Sex, male	297 (59.4%)
Patient type	
Surgical	200 (40%)
Medical	300 (60%)
APACHE II score at admission	18 (15–21)
McCabe score	
Nonfatal disease	190 (38%)
Ultimately fatal disease	77 (15.4%)
Rapidly fatal disease	233 (46.6%)
Reason for ICU admission	
Infection	122 (24.4%)
Postoperative monitoring	87 (17.4%)
Trauma	83 (16.6%)
Neurological disease	72 (14.4%)
Pulmonary disease	68 (13.6%)
Cardiovascular disease	36 (7.2%)
Poisoning/Burns	32 (6.4%)
Renal failure	130 (35.1%)
ICU length of stay, days	19 (10–33)
Length of mechanical ventilation, days	15 (7–27)
Transfusion	222 (44.4%)
Sepsis	47 (9.4%)
Pressure ulcer	76 (15.2%)
HCAI	273 (54.6%)
VAE	207 (41.4%)
CLABSI	108 (21.6%)
CAUTI	31 (6.2%)
SSI	9 (1.8%)
Patient-to-nurse ratio	
1 patient per nurse	79 (15.8%)
2 patients per nurse	104 (20.8%)
>2 patients per nurse	317 (63.4%)
Death	175 (35%)

IQR, Interquartile range; HCAI, Health Care-Associated Infection; VAE, Ventilator-Associated Event; CLABSI, Central Line-Associated Bloodstream Infection; CAUTI, Catheter-Associated Urinary Tract Infection; SSI, Surgical Site Infection

**Table 2.** The median ICU cost by category

**Cost categories**	**Cost per patient (€) Median (IQR)**	**Total cost (€)**	**Percentage of total cost (%)**
Fixed	2,592.5 (1,417.0–4,534.1)	1,741,486.4	27.5%
Variable	4,636.1 (1,448.7–10,685.2)	4,587,742.1	72.5%
Antibiotics	1,673.0 (442.1–7,301.2)	3,066,481.7	48.5%
Other drugs	1,432.9 (556.4–3,100.2)	1,324,274.7	20.9%
Blood products	0.0 (0.0–15.6)	8,781.6	0.1%
Medical consumables	98.8 (52.9–212.8)	91,748.8	1.5%
Diagnostic tests	134.7 (44.1–281.6)	96,455.3	1.5%
Total cost	7,756.8 (3,272.6–16,095.0)	6,329,228.3	

**Table 3.** ICU hospitalization cost by patient groups

**Variable**	**N**	**Cost, €**		**p-value**
		**Median**	**IQR**	
Age, years				0.709
<61	241	7.577,9	11.208,6	
≥61	259	7.979,1	14.117,0	
Sex				0.005
Female	203	5.740,0	11.336,8	
Male	297	8.887,0	13.180,2	
Patient type				0.402
Surgical	200	7.998,6	13.695,0	
Medical	300	7.657,0	11.828,4	
APACHE II score				<0.001
<18	230	5,483.2	9,341.4	
≥18	270	9,717.9	16,009.8	
Ultimately fatal or rapidly fatal disease				0.009
(McCabe score)				
Yes	310	8.572,0	11.265,0	
No	190	5.429,4	14.308,4	
Reason for ICU admission				
Infection	122	8.982,5	10.669,7	0.039
Postoperative monitoring	87	3.244,3	8.267,2	<0.001
Trauma	83	10.153,7	12.267,0	0.002
Neurological disease	72	6.878,9	12.013,5	0.758
Pulmonary disease	68	7.034,3	11.193,3	0.885
Cardiovascular disease	36	4.809,2	18.769,5	0.815
Poisoning/Burns	32	10.298,2	16.807,5	0.264
Renal failure				<0.001
Yes	130	11.723,1	19.801,0	
No	370	6.326,2	11.071,0	
Transfusion				<0.001
Yes	222	11.838,3	16.991,3	
No	278	4.715,2	8.951,2	
Sepsis				0.003
Yes	47	12.167,4	18.930,0	
No	453	7.350,6	11.658,7	
Pressure ulcer				<0.001
Yes	76	20.975,8	27.371,4	
No	424	6.432,0	9.625,5	
HCAI				<0.001
Yes	273	12.416,7	16.649,2	
No	227	3.428,6	5.685,4	
VAE				<0.001
Yes	207	12.167,4	16.073,1	
No	293	4.295,8	8.883,0	
CLABSI				<0.001
Yes	108	14.468,7	19.763,1	
No	392	6.104,2	10.623,2	
CAUTI				<0.001
Yes	31	21.483,5	40.215,8	
No	469	7.378,9	11.375,9	
SSI				0.030
Yes	9	12.537,0	22,866,7	
No	491	7.577,8	12.415,7	
Length of ICU stay, days				<0.001
<19	238	3,239.0	3,727,1	
≥19	262	14,015,5	16,469.6	
Length of mechanical ventilation, days				<0.001
<15	244	3,262.6	3,748.8	
≥15	256	14,069.1	16,571.5	
>2 patients per nurse				<0.001
Yes	317	10.104,8	15.869,4	
No	183	4.745,9	7.577,0	
Death				<0.001
Yes	175	9.676,8	18.133,0	
No	325	6.665,6	10.924,2	

IQR, Interquartile Range; HCAI, Health Care-Associated Infection; VAE, Ventilator-Associated Event; CLABSI, Central Line-Associated Bloodstream Infection; CAUTI, Catheter-Associated Urinary Tract Infection; SSI, Surgical Site Infection

**Table 4.** Univariate and Multivariate Analysis of Factors Impacting ICU hospitalization Costs Dependent variable: natural logarithm of ICU hospitalization cost, N=500, Adjusted R2 = 67.3%

**Univariable Analysis**	**B**	**95% CI**	**Exp(B)**
Female sex	−0.261	−0.450 to −0.072	0.77
Admission APACHE II Score	0.043	0.025 to 0.060	1.043
Rapidly or ultimately fatal disease	0.285	0.094 to 0.476	1.330
Infection as reason for ICU admission	0.245	0.028 to 0.461	1.278
Postoperative monitoring admission	−0.678	−0.904 to −0.451	0.508
Trauma as reason for ICU admission	0.573	0.314 to 0.831	1.773
Pressure ulcers	1.105	0.863 to 1.346	3.019
Sepsis	0.470	0.153 to 0.788	1.60
Renal failure	0.544	0.336 to 0.751	1.723
Blood transfusion	0.774	0.599 to 0.950	2.168
ICU length of stay	0.040	0.037 to 0.043	1.041
Mechanical ventilation duration	0.043	0.039 to 0.046	1.043
HCAI	1.197	1.042 to 1.353	3.310
VAE	0.920	0.748 to 1.092	2.510
CLABSI	0.843	0.628 to 1.057	2.323
CAUTI	1.018	0.641 to 1.396	2.768
SSI	0.769	0.069 to 1.469	2.158
Patient-to-nurse ratio >2	0.617	0.431 to 0.803	1.853
Death	0.400	0.207 to 0.593	1.492
**Multivariable Analysis**	**B**	**95% CI**	**Exp(B)**
Female sex	−0.026	−0.138 to 0.086	0.974
Admission APACHE II Score	0.012	0.000 to 0.024	1.012
Infection as reason for ICU admission	0.092	−0.062 to 0.246	1.096
Postoperative monitoring admission	−0.083	−0.244 to 0.079	0.920
Trauma as reason for ICU admission	0.143	−0.020 to 0.306	1.154
Pressure ulcers	0.131	−0.041 to 0.304	1.140
Sepsis	−0.105	−0.323 to 0.113	0.900
Renal failure	−0.029	−0.178 to 0.120	0.971
Blood transfusion	0.236	0.112 to 0.359	1.266
ICU length of stay	0.033	0.024 to 0.041	1.034
Mechanical ventilation duration	−0.001	−0.011 to 0.008	0.999
VAE	0.267	0.144 to 0.390	1.306
CLABSI	0.217	0.063 to 0.372	1.242
CAUTI	−0.171	−0.415 to 0.073	0.843
SSI	0.616	0.210 to 1.022	1.852
Patient-to-nurse ratio >2	0.497	0.372 to 0.621	1.644
Death	0.082	−0.061 to 0.226	1.085

B, Regression coefficient, Exp(B), Exponentiated regression coefficient; CI, Confidence Interval; HCAI, Health Care-Associated Infection; VAE, Ventilator-Associated Event; CLABSI, Central Line-Associated Bloodstream Infection; CAUTI, Catheter-Associated Urinary Tract Infection; SSI, Surgical Site Infection.

Translating these findings into economic terms, and using the median ICU cost per patient (€7,756.8) as a reference, we estimated the financial impact of each independent predictor based on the corresponding Exp(B) values from the multivariable regression model. Patients who received blood transfusions incurred an average of approximately €2,063 more in ICU costs. Each additional day of ICU stay was associated with a 3.4% increase in cost, corresponding to about €264 per day. VAE and CLABSI led to excess costs of €2,374 and €1,877 per patient, respectively, while SSI nearly doubled the cost of care, adding an estimated €6,609. Furthermore, treatment in ICUs with a patient-to-nurse ratio greater than 2 was associated with a median cost increase of approximately €5,000 per patient.

## Discussion

This prospective, bottom-up micro-costing study provides a comprehensive assessment of ICU hospitalization costs and the key factors associated with cost variation, based on data from a representative sample of four multidisciplinary ICUs in the Attica region of Greece. While micro-costing analyses exist for high-resource systems, their application in resource-constrained contexts like Greece is scarce [9,22]. The analysis, with its granular patient-level data, was designed to provide the level of accuracy critical for optimizing resources in such settings. The dataset, encompassing 500 patients over a two-year period and more than 12,000 ICU patient-days, provides valuable insight into both the clinical and operational determinants of cost. With a median cost of €7,756.8 per ICU stay, the financial burden of critical care is considerable, especially when factoring in those variable costs comprised nearly 73% of the total expenditure, with antibiotics alone accounting for nearly half of these costs. This high proportion likely reflects the frequent use of broad-spectrum and combination antimicrobial therapies in critically ill patients, particularly in settings with a high prevalence of multidrug-resistant organisms. Data from the European Centre for Disease Prevention and Control indicate that Greece has among the highest levels of antimicrobial resistance in European acute care hospitals [15]. These elevated antibiotic-related costs are driven not only by endemic multidrug-resistant pathogens, but also by local prescribing practices. Previous reports highlight significant variability in the implementation of, and engagement with, antimicrobial stewardship programs across Greek hospitals due to resource constraints [23,24]. Taken together, these findings underscore the importance of strengthening antimicrobial stewardship and infection prevention strategies in Greek ICUs, as these interventions may not only improve clinical outcomes but also reduce unnecessary healthcare expenditures [25].

The multivariable regression analysis identified several independent predictors of increased ICU cost. Blood transfusion emerged as one of the most prominent contributors, associated with a 26.6% increase in cost, translating to an average additional expense of approximately €2,060 per patient. This aligns with existing literature highlighting transfusion as a high-cost intervention, often linked with longer ICU stays and higher morbidity [26,27].

Similarly, ICU length of stay demonstrated a strong and consistent association with cost, with each additional day increasing overall cost by approximately 3.4%, or €264 per day, emphasizing the cumulative financial impact of prolonged critical care. Supporting these findings, a study in Turkey demonstrated that cost per ICU day decreases after the first few days, yet total expenditures remain directly proportional to length of stay [28]. Likewise, a Canadian study found that marginal cost savings were greatest when ICU length of stay was reduced to under four days, further underscoring the financial implications of extended stays [29]. A larger European prospective micro-costing study in 23 French hospitals reported mean daily ICU costs of €1,425 ± €520, with daily costs correlated with severity of illness and nursing time [22]. These studies collectively affirm that cumulative ICU days significantly escalate ICU costs—both due to high-intensity early days and the additive effect of extended resource use. Thus, minimizing ICU length of stay via structured care protocols, early mobilization, and multidisciplinary discharge planning may substantially reduce overall ICU costs without compromising clinical outcomes.

Device-associated infections, particularly VAE and CLABSI, were also shown to substantially increase hospitalization costs. The occurrence of VAE was associated with a 30.6% increase in costs (€2,374), while CLABSI was associated with a 24.2% increase (€1,880). These results reflect similar patterns reported in both international and Greek studies, where device-associated infections not only prolong length of stay but also increase diagnostic, pharmaceutical, and procedural expenditures [30,31,32,33,34]. In particular, the financial impact of CLABSI has been estimated to range from €13,727 to $23,619 [32,33,34], while the additional cost of VAE ranges from €3,227 to $6,775 [29,30], highlighting the widely recognized economic burden of these infections.

Alongside these device-associated infections, SSI were also identified as a significant independent predictor of increased ICU costs in the multivariate analysis, indicating that SSI nearly doubled the cost of ICU care. This substantial cost increase, estimated at approximately €6,609 per patient, further underscores the severe economic burden posed by infections in critical care settings. These findings align with previous studies highlighting that SSI prolong hospitalization, increase the need for additional interventions, and substantially raise healthcare costs [35,36]. Taken together, these results emphasize the critical importance of infection prevention strategies targeting both device-associated infections and SSI, as effective control measures could yield meaningful cost savings alongside improved patient outcomes.

A striking result of this study was the strong association between higher patient-to-nurse ratios and increased costs. Patients treated in ICUs where nurses cared for more than two patients each had 64.4% higher ICU costs, equating to an approximate excess of €5,000 per admission. While higher staffing levels imply greater upfront human resource costs, existing evidence suggests they may actually reduce overall expenditures by preventing complications, shortening ICU length of stay, and improving patient outcomes [37,38]. Notably, several variables that were significant in univariate analysis, including sepsis, renal failure, trauma, pressure ulcers, and mechanical ventilation duration, lost significance in the multivariable model. This suggests that these factors may increase ICU costs indirectly by acting as mediators of prolonged hospitalization or increased risk of nosocomial infections. For instance, patients with sepsis often require extended mechanical support, leading to higher rates of VAE and thus longer ICU stays [39,40]. Similar reasoning applies to pressure ulcers, which may not only be indicators of poor functional status but also predictors of extended hospitalization and secondary infections [41].

These findings have several important implications. First, they underline the critical importance of infection prevention and control programs. Interventions that reduce the incidence of CLABSI, VAE, and SSI could yield substantial cost savings, in addition to improving patient outcomes. Second, they highlight the potential cost-effectiveness of evidence-based transfusion strategies that avoid unnecessary blood product administration. Third, they reinforce the economic rationale for maintaining adequate nurse staffing levels in the ICU, a strategy that is often challenged during periods of resource limitation but which may ultimately reduce rather than increase total healthcare spending.

Despite the robustness of these findings, certain limitations must be acknowledged. Although the study included multiple ICUs, all were located within the Attica region of Greece, and therefore reflects a specific public sector tertiary care context. As a result, caution is warranted when generalizing the findings to private-sector ICUs, smaller regional hospitals, or healthcare systems with different financing structures, staffing models, or infection epidemiology. In particular, the high burden of antimicrobial resistance and staffing constraints observed in Greece may limit comparability with higher-resource settings. Furthermore, the analysis was conducted from the hospital perspective and included only direct ICU costs; therefore, indirect and societal costs such as post-ICU hospitalization or productivity losses, were not considered. Additionally, although a wide range of clinical and organizational variables were examined, unmeasured confounders such as ICU-specific protocols, staffing experience, or case-mix complexity may still have influenced the results. Further research should involve multi-center studies in diverse settings to validate these findings and expand the cost analysis to include indirect and societal perspectives.

## Conclusion

In conclusion, the findings of this study reveal that ICU hospitalization costs are significantly influenced by both patient-related clinical factors and structural elements of ICU organization. Infections acquired during ICU stay—particularly VAE, CLABSI, and SSI—emerged as independent predictors of increased costs. Additionally, blood transfusion and prolonged ICU length of stay were strongly associated with higher expenditure. Importantly, the study also identified organizational determinants such as a high patient-to-nurse ratio as a significant cost driver, highlighting the complex interplay between resource availability, care delivery, and economic outcomes in intensive care settings. These results underscore the importance of adopting a multifaceted approach to cost containment in the ICU. Investing in effective infection prevention strategies and maintaining safe staffing levels could not only reduce complications but also alleviate the financial burden on healthcare systems. Efforts should also focus on optimizing ICU workflows to minimize unnecessary prolongation of stay and avoid costly interventions. Policymakers and hospital administrators should consider that improving quality of care—through prevention, early intervention, and organizational support—can lead to both better clinical outcomes and more efficient resource use.

## Authors’ contribution

AK (Conceptualization; Data curation; Formal analysis; Investigation; Methodology; Project administration; Resources; Validation; Visualization; Writing – original draft; Writing – review & editing)

MK (Methodology; Resources; Validation; Writing – review & editing)

CS (Investigation; Methodology; Resources; Visualization; Writing – original draft)

PM (Conceptualization; Methodology; Supervision; Writing – review & editing)
